# PEPlife: A Repository of the Half-life of Peptides

**DOI:** 10.1038/srep36617

**Published:** 2016-11-07

**Authors:** Deepika Mathur, Satya Prakash, Priya Anand, Harpreet Kaur, Piyush Agrawal, Ayesha Mehta, Rajesh Kumar, Sandeep Singh, Gajendra P. S. Raghava

**Affiliations:** 1 Bioinformatics Centre, CSIR-Institute of Microbial Technology, Chandigarh, India

## Abstract

Short half-life is one of the key challenges in the field of therapeutic peptides. Various studies have reported enhancement in the stability of peptides using methods like chemical modifications, D-amino acid substitution, cyclization, replacement of labile aminos acids, etc. In order to study this scattered data, there is a pressing need for a repository dedicated to the half-life of peptides. To fill this lacuna, we have developed PEPlife (http://crdd.osdd.net/raghava/peplife), a manually curated resource of experimentally determined half-life of peptides. PEPlife contains 2229 entries covering 1193 unique peptides. Each entry provides detailed information of the peptide, like its name, sequence, half-life, modifications, the experimental assay for determining half-life, biological nature and activity of the peptide. We also maintain SMILES and structures of peptides. We have incorporated web-based modules to offer user-friendly data searching and browsing in the database. PEPlife integrates numerous tools to perform various types of analysis such as BLAST, Smith-Waterman algorithm, GGSEARCH, Jalview and MUSTANG. PEPlife would augment the understanding of different factors that affect the half-life of peptides like modifications, sequence, length, route of delivery of the peptide, etc. We anticipate that PEPlife will be useful for the researchers working in the area of peptide-based therapeutics.

Peptide therapeutics has become a major field of biomedical and pharmaceutical research^1^. The underlying reason is that the peptides as therapeutic agents are better than the chemical drugs in providing greater safety, target specificity and potency^1^,^2^,^3^,^4^,^5^. The peptides have reduced side effects and do not accumulate in the body. Nowadays peptides are being used as drugs in case of different diseases such as multiple sclerosis, prostate cancer, endometriosis, acromegaly, etc.^1^. Peptides containing various therapeutic properties have been discovered^6^,^7^,^8^,^9^,^10^,^11^,^12^,^13^,^14^,^15^,^16^,^17^,^18^,^19^,^20^ and their number is increasing with time^21^. Owing to their applicability, a number of bioinformatics platforms have been developed to assist peptide therapeutics^22^,^23^,^24^,^25^,^26^,^27^,^28^. According to a recent report, 128 peptides are in the clinical pipeline. Of these 128 peptides, 74 are in Phase II and 14 in Phase III clinical trials^29^. Peptides like INGAP for diabetes, N-acetyl-aspartyl-glutamate for geriatric depression and GO-203–2C, p28 (CPP) and CDCA1 for cancer treatment are in clinical trials (https://clinicaltrials.gov). Inspired by the potential applications, peptide therapeutics have been projected as a billion dollar market^4^,^5^. For example only in the year 2011, United States approved 25 therapeutic peptides that had global sales of 14.7 billion US dollars^29^.

Despite numerous advantages, there are a few challenges associated with therapeutic peptides that obstruct the way of the maximal benefits of peptides. These challenges include high production cost, low storage stability and suboptimal *in vivo* half-life^5^. The technological developments promise to increase the production and the storage stability^4^,^5^. The suboptimal *in vivo* half-life remains a challenge as the short half-life of a peptide reduces its bioavailability that is required for its optimal function^30^. Thus, it is imperative to focus on designing of peptides with optimal half-life to ensure their optimal action.

In order to address this important topic, a large number of experimental studies have been dedicated to improve and optimize the half-life of peptides^31^,^32^,^33^,^34^. Although the data from these studies are very useful, they are scattered in the literature and therefore, are difficult to access and use. With the purpose of providing assistance to the scientific community, in this study an attempt has been made to develop a platform ‘PEPlife’ to provide data related to the half-life of peptides at a single source. We have also incorporated various tools and modules in PEPlife to assist users in searching, comparing and analyzing the peptides, their half-lives and the related details. We hope that PEPlife will be helpful for the scientific community to design peptides with optimal stabilities.

## System and Methods

### Data Collection

The data was manually collected and curated from published research articles and patents. Only those peptides were included in the database, whose half-life was experimentally determined. We queried PubMed to search for research articles and The Lens for patents. The query ‘(peptide[Title/Abstract] AND half-life[Title/Abstract])’ was used to retrieve articles relevant to half-life of peptides from PubMed. It resulted in ~2280 articles as on November 2015. During the initial screening, the articles lacking relevant information and reviews were excluded. Around 900 potential papers were scrutinized to mine the required fields. Finally, data was systematically curated from 335 articles. Similarly, full-text of granted patents were obtained from The Lens and manually screened to filter the patents with relevant information for data curation. We also collected relevant information about FDA approved peptide drugs from DrugBank^35^ and related literature.

In PEPlife, we have systematically compiled comprehensive information about each peptide. The information includes the peptide’s name, sequence, length, terminal and non-terminal modifications, biological property, assay used to determine the half-life of peptide. To maintain complete information, we made multiple entries for the same peptide if its bioactivity or half-life was tested using different concentrations, conditions, routes of administration, etc. This complete information, thus, highlights the influence of these subtle conditions on the half-life of peptides.

### Database Architecture and Web Interface

**Figure 1:**
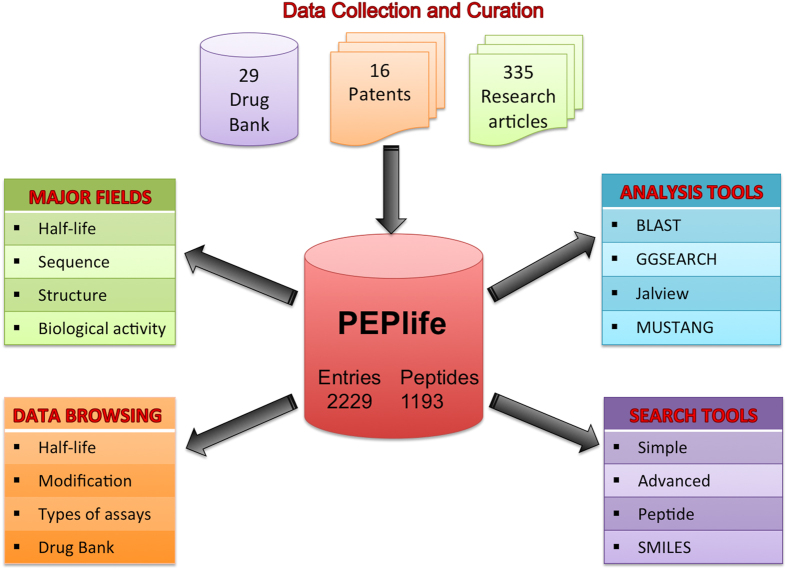
Architecture of PEPlife database.

PEPlife was built using Apache HTTP server on Linux Platform. MySQL an object-relational database management system (RDBMS) was used to manage all the data in the backend. It allows easy retrieval and storage of the data in the database. HTML, CSS, PHP and JavaScript were used to develop the front-end web interface. The architecture of PEPlife is represented in Fig. 1.

### Database Content

The information in PEPlife can be categorized into two broad types: primary information and secondary information. The primary information has been curated from the literature and consists of the following major fields namely: (i) PMID, (ii) the peptide sequence, (iii) the name of peptide, (iv) the length of peptide, (v) N-terminal modification, (vi) C-terminal modification (vii) configuration (linear or cyclic) (viii) chirality of the amino acids, (ix) chemical modification, (x) origin of the peptide, (xi) biological activity of the peptide, (xii) half-life, (xiii) assay types, (xiv) sample on which the half-life was tested and (xv) Patent ID.

The secondary information, which was derived from the primary information, includes the tertiary structures of peptides. To obtain the structural information, all the peptides of PEPlife database were searched and mapped to the peptide sequences in the Protein Data Bank (PDB)^36^. After obtaining the exact sequence match in PDB, the structure same as the match was assigned to the query peptide. Using this approach, we determined the structures of 265 peptides. In the cases where identical peptide sequences were not available, we predicted the structures of the peptides using PEPstrMOD^37^ which is updated and advanced version of PEPstr^38^, containing natural and modified residues^38^,^39^,^40^,^41^. Due to the unavailability of force-field libraries for complex chemical modifications (e.g., pegylation, penicillamine, etc.), the structures of the peptides containing such modifications were not predicted. The structures of a total of 36 peptides, which had amino acids lesser than five residues, could not be predicted by using the software mentioned above. Therefore, in this case where a peptide has less than five residues, we used a linear conformation with dihedral angles (ϕ and ψ) as 180°. The initial structure was subjected to energy minimization and molecular dynamics simulation. The trajectory of the whole simulation was searched for the conformation that had minimum energy. The minimum-energy-structure was considered as the final predicted structure. The structures of 132 peptides having more than 40 residues were predicted using I-TASSER web service^42^. The tertiary structures of the peptide entries from DrugBank were also predicted in the same way. Out of 29 peptide entries from DrugBank, 6 entries were mapped to PDB, 9 entries were predicted using PEPstrMOD, and the rest 14 entries with modified residues were not predicted.

The secondary structures of all peptides were assigned using DSSP software from their tertiary structure^43^. DSSP assigns the secondary structure into eight different states (B: beta-bridge; C: loop; E: extended strand; G: 3/10 helix; H: alpha-helix; I: pi-helix; S: bend and T: turn). The secondary structure analysis of the predicted structures (except DrugBank peptides) revealed that the peptide residues frequently belong to loop regions (~32%), followed by helix (~30%), turns (~17%) and bends (~19%). Only a few peptide residues were observed in strand regions (~2%). The predicted peptide structures were also converted into SMILES notation using Open Babel software^44^.

### Implementation of Web Tools

A number of tools have been integrated for data retrieval, similarity search and data analysis; following is the brief description of different options available in PEPlife.

### Search Tools

We have incorporated 4 different modules under the Search option to facilitate easy retrieval of data: Simple, Advanced, Peptide and SMILES. In the Simple Search, the website facilitates users to search peptides according to any of the fields in the database. In this option, users can also select the fields to display in the results. In the case of the Advanced Search, users can perform complex and multiple queries for extracting desired entries from the database. This option allows the use of standard logical operators (“=”, “>”, “<” and “LIKE”). A user can combine the outputs of different queries using operators like “AND & OR”. The Peptide Search tool searches exact as well as substring matches of a given peptide sequence among the peptide sequences available in PEPlife. We have also maintained structures of the peptides in SMILES format to assist users to understand the property of peptides at atom/bond level. The SMILES Search facilitates users to search a query peptide in SMILES format present in our database.

### Browsing Tools

In PEPlife, we have provided a simple yet thorough class-wise browsing facility, in which all the peptide-entries have been categorized into different classes. In this module, the information related to a peptide can be browsed using following seven criteria (i) Half-life, (ii) Organism and Media (where the half-life was tested), (iii) Peptide Length (iv) Publication Year (v) Type of Modification (vi) Type of Assay (used to detect the half-life) and (vii) DrugBank peptides.

### Analysis Tools

PEPlife is studded with a number of web-based tools for performing various sequence and structure analyses of query peptides. BLAST, Smith-Waterman and GGSEARCH tools allow the user to perform similarity search of their query peptide against PEPlife database. The Peptide Mapping tool facilitates users to perform sub-search and super-search against PEPlife peptides. The Sequence Alignment tool allows the user to align query peptide with only user-selected peptides from PEPlife by using their PEPlife-IDs. The Structure Alignment tool aligns the structure of query peptide with the structure of the chosen peptide from PEPlife. The PDB file of the query peptide and PEPlife-ID of the subject peptide is submitted to perform the structural alignment.

### Data Statistics

PEPlife harbors total 2229 entries containing relevant information about 1193 unique peptides and their half-lives. 2066 entries were collected from 335 published articles, 134 entries from 16 patents and 29 entries of from DrugBank. A significant portion of the entries (~833 entries) belongs to the period from the year 2005 to the year 2009. These peptides have been reported to show a diversity of biological behaviors, such as anticancer, antiviral, antimicrobial, antibacterial, neurotransmitter, erythropoiesis-stimulant, anticoagulant, antihyperglycemic, insulinotropic, antihypertensive, etc.

**Figure 2:**
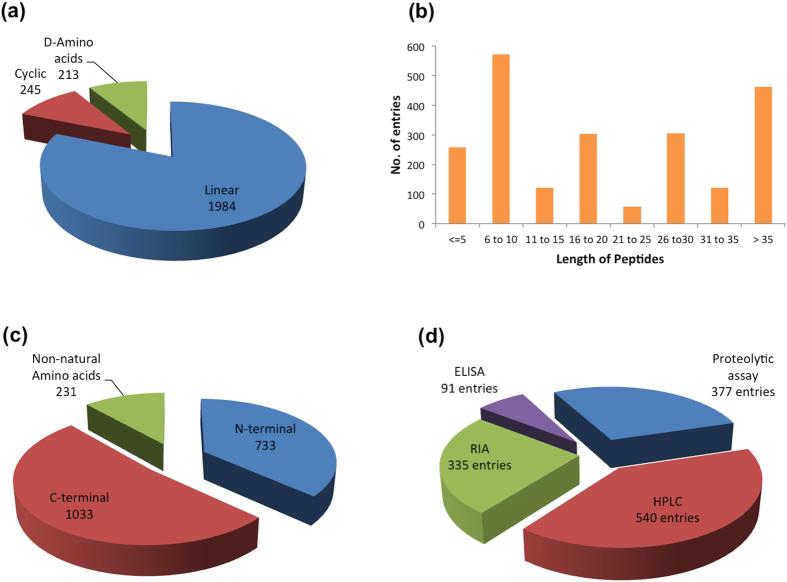
Distribution of peptides based on **(a**) conformation and configuration of amino acids, (**b**) length, **(c)** modifications and (**d**) assays used to measure half-life.

**Figure 3:**
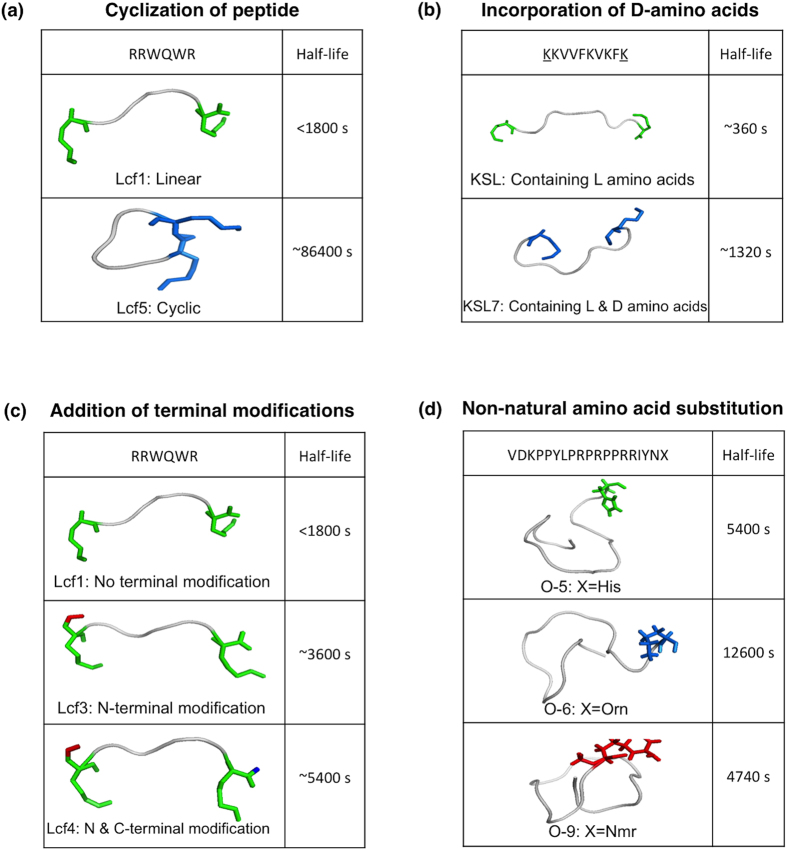
Examples to show the effects of modifications on the half-life of peptide analogues. (**a**) Cyclization of peptides-The half-life of Lcf1 (RRWQWR) increases on head to tail cyclization of the same sequence in its analogue Lcf5. (**b**) Incorporation of D-amino acids- KSL7 (kKVVFKVKFk) with 2 D-amino acids has a longer half-life than KSL (KKVVFKVKFK). (**c**) Addition of terminal modifications- Lcf3 (CH_3_CO-RRWQWR) with N-terminal modification and Lcf4 (CH_3_CO-RRWQWR-NH_2_) with both N & C-terminal modifications have longer half-life than Lcf1 (RRWQWR) with no terminal modifications. (**d**) Non-natural amino acid substitution- O-6 (VDKPPYLPRPRPPRRIYN-Orn) and O-9 (VDKPPYLPRPRPPRRIYN-Nmr) with non-natural amino acids have longer half-life than O-5 (VDKPPYLPRPRPPRRIYNH).

Peptides in PEPlife have different conformations, amino acid configurations and lengths (Fig. 2A). The examples of the effects of modifications on the half-life of peptides are given in Fig. 3 and Supplementary Table S1. Peptides in 245 entries are cyclic. The peptides in 213 entries have peptides with mixed (i.e., containing both L and D) amino acid configurations. Lengths of the peptides vary from less than five amino acids to more than 35 amino acids. Peptides with the lengths from six to ten amino acids have the maximum number of entries (571 entries), followed by the peptides having >35 amino acids (462 entries). Peptides composed of 21 to 25 amino acids are present in the least number of entries (57 entries) (Fig. 2B).

In order to increase the half-life of peptides, various modifications have been incorporated in the peptides. Among them, most of the modifications have been done at the termini (Fig. 2C). The maximal number of N-terminal modifications include the addition of 2,4-dichlorophenoxyacetic acid with (CH2)_n_-spacers, followed by acylation. Besides, PEGylation, glycosylation, succinylation, addition of human serum albumin (HSA) and hydroxylation have been utilized as important N-terminal modifications to improve the half-life of peptides. Amidation is the most used C-terminal modification followed by biotinylation with PEG (polyethylene glycol) spacers. The other C-terminal modifications include additions of Human serum albumin (3 entries), cholesterol (2 entries), PEG (24 entries), XTEN (4 entries), Fc-region (14 entries), etc. These non-terminal modifications include methylation (27 entries), addition of fatty acid chains (7 entries), addition of carbohydrate chains (2 entries), reduced amide bonds (33 entries) and reduced carbamate bonds (16 entries) etc. and incorporation of non-natural amino acids like biphenylalanine (Bip) (36 entries), pyroglutamic acid (pGlu) (27 entries), sarcosine (Sar) (45 entries), ornithine (Orn) (12 entries), norleucine (Nle) (4 entries), etc.

The entries in PEPlife show a number of *in vivo* (948 entries) and *in vitro* (1265 entries) methods used to assess the half-life of peptides. These methods include mass spectrometry, immunoassays, radiolabeling, spectroscopy and various other assays. Some of the favored assessment methods include HPLC (540 entries), radioimmunoassay (335 entries) and ELISA (91 entries) (Fig. 2D).

## Discussion

The half-life of a peptide determines its bioavailability to the organism; a peptide having therapeutic advantages should also possess optimal bioavailability to be used as a drug. The short half-life of a therapeutic peptide can lead to the less bioavailability. Despite the significant relevance of half-life in bioavailability, so far no platform is available which covers a broad variety of information related to the half-life of peptides. However, few bioinformatics platforms predict the half-life of specific peptides in the specific environments only^31^,^45^. Moreover, these platforms do not contain a wide range of information. Therefore, it is evident that there is a need for a database that has a broad scope and usage in peptide half-life improvement. In this report, we have created a database as an attempt to fulfill the lacuna and to provide a repertoire of information related to the half-life of peptides having a variety of properties and modifications. The database also covers the variations observed in the half-life according to different environments, organisms and different routes of administration.

The half-life of a peptide depends on both, the organism and the peptide. A number of *in vitro* and *in vivo* studies have been done to understand the relationship between the half-life of peptides and their sequences, structures, modifications; host organisms; and drug administration routes in the host organism. The factors which affect the enzymatic degradation and the pharmacokinetics of a peptide in an organism play crucial roles in deciding the stability of that peptide^46^,^47^. Apparently the factors that lower the enzymatic degradation and metabolism of a peptide tend to stabilize the peptide^48^,^49^. Different organisms have different pharmacokinetics and different extent of proteolysis of a peptide, leading to a difference in its half-life^46^,^47^. Moreover, different individuals of the same species can have variable pharmacokinetics of the same peptide, leading to the variable half-life of the peptide^47^.

The significant factors affecting half-life include the sequence of a peptide, modifications, administration routes, and the amount of the peptide (dose). It is observed that the sequence variants of a peptide have different half-lives. Chemical modifications also alter the half-life^49^. To achieve improved half-life, the inclusion of chemical modifications such as the use of D-amino acids, non-natural amino acids (e.g., ornithine), PEGylation and N and C-terminal modifications have been extensively employed. A peptide administered in different organisms via the same route has different half-lives^49^. Furthermore, different administration routes also affect the half-life of a peptide^50^. Clearly, all the mentioned details are necessary to improve the half-life of therapeutic peptides. For this reason, it is essential to store such details at one platform for their easy access and use.

PEPlife is a repository of valuable information related to the stability of peptides. It harbors extensive and systematic cataloging of the data related to the half-life of peptides and the affecting factors. This information can prove indispensable for the rational design of peptides of therapeutic importance. To add further advantages, a number of tools have been provided in the database to facilitate the extraction and analysis of the compiled information. We anticipate that PEPlife will be helpful not only to satisfy half-life queries but also to understand the properties of peptides that govern their half-lives.

In the future, various interesting studies can be done using the data of PEPlife. Some of them can be as follows: (i) structures available in PEPlife can be used for docking and various membrane simulations studies, (ii) the dataset of PEPlife can be used for development of various prediction methods for peptide half-life, and, (iii) the SMILES of PEPlife can be used to develop QSAR models. We hope that PEPlife will be a useful resource for researchers working in the area of designing of therapeutic peptides.

### Update of PEPlife

We will update PEPlife at regular intervals to further widen the coverage of half-life of peptides reported in literature. PEPlife also provides the users an option to submit new entries of peptides and their half-life on its web interface by filling an HTML form. Our team will confirm the validity of each new entry before incorporating into PEPlife in order to maintain a high level of quality.

### Limitations

We have made an attempt to cover as much information as possible related to half-life of peptides by manual curation, though it is possible that a few articles might not be incorporated that could not be fetched with our search criteria. We have provided structural information of most of the peptides but due to unavailability of force-field libraries of complex modification of peptides, a few structures of peptides could not be predicted.

## Additional Information

**How to cite this article**: Mathur, D. *et al.* PEPlife: A Repository of the Half-life of Peptides. *Sci. Rep.* **6**, 36617; doi: 10.1038/srep36617 (2016).

**Publisher’s note:** Springer Nature remains neutral with regard to jurisdictional claims in published maps and institutional affiliations.

## Supplementary Material

Supplementary Information

## References

[b1] Craik D. J., Fairlie D. P., Liras S. & Price D. The future of peptide-based drugs. Chem Biol Drug Des 81, 136–147, doi: 10.1111/cbdd.12055 (2013). 23253135

[b2] Holohan C., Van Schaeybroeck S., Longley D. B. & Johnston P. G. Cancer drug resistance: an evolving paradigm. Nat Rev Cancer 13, 714–726, doi: 10.1038/nrc3599 (2013). 24060863

[b3] Vlieghe P., Lisowski V., Martinez J. & Khrestchatisky M. Synthetic therapeutic peptides: science and market. Drug Discov Today 15, 40–56, doi: 10.1016/j.drudis.2009.10.009 (2010). 19879957

[b4] Lax R. The future of peptide development in the pharmaceutical industry. PharManufacturing: The international peptide review 2, 10–15 (2010).

[b5] Sun L. Peptide-based drug development. Mod. Chem. Appl 1 (2013).

[b6] Wang G., Li X. & Wang Z. APD2: the updated antimicrobial peptide database and its application in peptide design. Nucleic Acids Res 37, D933–D937, doi: 10.1093/nar/gkn823 (2009). 18957441 PMC2686604

[b7] Waghu F. H. *et al.* CAMP: Collection of sequences and structures of antimicrobial peptides. Nucleic Acids Res. 42, D1154–D1158, doi: 10.1093/nar/gkt1157 (2014). 24265220 PMC3964954

[b8] Qureshi A., Thakur N., Tandon H. & Kumar M. AVPdb: a database of experimentally validated antiviral peptides targeting medically important viruses. Nucleic Acids Res. 42, D1147–D1153, doi: 10.1093/nar/gkt1191 (2014). 24285301 PMC3964995

[b9] Mehta D. *et al.* ParaPep: a web resource for experimentally validated antiparasitic peptide sequences and their structures. Database (Oxford) 2014, doi: 10.1093/database/bau051 (2014). PMC4054663 24923818

[b10] Hammami R., Ben Hamida J., Vergoten G. & Fliss I. PhytAMP: a database dedicated to antimicrobial plant peptides. Nucleic Acids Res 37, D963–D968, doi: 10.1093/nar/gkn655 (2009). 18836196 PMC2686510

[b11] Seshadri Sundararajan V. *et al.* DAMPD: a manually curated antimicrobial peptide database. Nucleic Acids Res 40, D1108–D1112, doi: 10.1093/nar/gkr1063 (2012). 22110032 PMC3244992

[b12] Piotto S. P., Sessa L., Concilio S. & Iannelli P. YADAMP: yet another database of antimicrobial peptides. Int J Antimicrob Agents 39, 346–351, doi: 10.1016/j.ijantimicag.2011.12.003 (2012). 22325123

[b13] Gautam A. *et al.* CPPsite: a curated database of cell penetrating peptides. Database (Oxford) 2012, bas015, doi: 10.1093/database/bas015 (2012). 22403286 PMC3296953

[b14] Wynendaele E. *et al.* Quorumpeps database: chemical space, microbial origin and functionality of quorum sensing peptides. Nucleic Acids Res 41, D655–D659, doi: 10.1093/nar/gks1137 (2013). 23180797 PMC3531179

[b15] Van Dorpe S. *et al.* Brainpeps: the blood-brain barrier peptide database. Brain Struct Funct 217, 687–718, doi: 10.1007/s00429–011–0375–0 (2012). 22205159

[b16] Théolier J., Fliss I., Jean J. & Hammami R. MilkAMP: a comprehensive database of antimicrobial peptides of dairy origin. Dairy Science & Technology 94, 181–193 (2014).

[b17] Novkovic M., Simunic J., Bojovic V., Tossi A. & Juretic D. DADP: the database of anuran defense peptides. Bioinformatics 28, 1406–1407, doi: 10.1093/bioinformatics/bts141 (2012). 22467909

[b18] Zhao X., Wu H., Lu H., Li G. & Huang Q. LAMP: A Database Linking Antimicrobial Peptides. PLoS One 8, e66557, doi: 10.1371/journal.pone.0066557 (2013). 23825543 PMC3688957

[b19] Agrawal P. *et al.* CPPsite 2.0: a repository of experimentally validated cell-penetrating peptides. Nucleic Acids Res 44, D1098–D1103, doi: 10.1093/nar/gkv1266 (2016). 26586798 PMC4702894

[b20] Tyagi A. *et al.* CancerPPD: a database of anticancer peptides and proteins. Nucleic Acids Res 43, D837–D843, doi: 10.1093/nar/gku892 (2015). 25270878 PMC4384006

[b21] McGregor D. P. Discovering and improving novel peptide therapeutics. Curr Opin Pharmacol 8, 616–619, doi: 10.1016/j.coph.2008.06.002 (2008). 18602024

[b22] Dhanda S. K. *et al.* Novel in silico tools for designing peptide-based subunit vaccines and immunotherapeutics. Brief Bioinform, doi: 10.1093/bib/bbw025 (2016). 27016393

[b23] Gautam A. *et al.* *In silico* approaches for designing highly effective cell penetrating peptides. J Transl Med 11, 74, doi: 10.1186/1479–5876–11–74 (2013). 23517638 PMC3615965

[b24] Sharma A. *et al.* Computational approach for designing tumor homing peptides. Sci Rep 3, 1607, doi: 10.1038/srep01607 (2013). 23558316 PMC3617442

[b25] Thakur N., Qureshi A. & Kumar M. AVPpred: collection and prediction of highly effective antiviral peptides. Nucleic Acids Res 40, W199–W204, doi: 10.1093/nar/gks450 (2012). 22638580 PMC3394244

[b26] Gupta S. *et al.* *In silico* approach for predicting toxicity of peptides and proteins. PLoS One 8, e73957, doi: 10.1371/journal.pone.0073957 (2013). 24058508 PMC3772798

[b27] Gautam A. *et al.* Hemolytik: a database of experimentally determined hemolytic and non-hemolytic peptides. Nucleic Acids Res 42, D444–D449, doi: 10.1093/nar/gkt1008 (2014). 24174543 PMC3964980

[b28] Chaudhary K. *et al.* A Web Server and Mobile App for Computing Hemolytic Potency of Peptides. Sci Rep 6, 22843, doi: 10.1038/srep22843 (2016). 26953092 PMC4782144

[b29] Kaspar A. A. & Reichert J. M. Future directions for peptide therapeutics development. Drug Discov Today 18, 807–817, doi: 10.1016/j.drudis.2013.05.011 (2013). 23726889

[b30] Jenssen H. & Aspmo S. I. Serum stability of peptides. Methods Mol Biol 494, 177–186, doi: 10.1007/978–1–59745–419–3_10 (2008). 18726574

[b31] Sharma A., Singla D., Rashid M. & Raghava G. P. Designing of peptides with desired half-life in intestine-like environment. BMC Bioinformatics 15, 282, doi: 10.1186/1471–2105–15–282 (2014). 25141912 PMC4150950

[b32] Knappe D., Henklein P., Hoffmann R. & Hilpert K. Easy strategy to protect antimicrobial peptides from fast degradation in serum. Antimicrob Agents Chemother 54, 4003–4005, doi: 10.1128/AAC.00300–10 (2010). 20585128 PMC2934954

[b33] Adessi C. *et al.* Pharmacological profiles of peptide drug candidates for the treatment of Alzheimer’s disease. J Biol Chem 278, 13905–13911, doi: 10.1074/jbc.M211976200 (2003). 12578830

[b34] Gong N. *et al.* Site-specific PEGylation of exenatide analogues markedly improved their glucoregulatory activity. Br J Pharmacol 163, 399–412, doi: 10.1111/j.1476–5381.2011.01227.x (2011). 21244372 PMC3087140

[b35] Law V. *et al.* DrugBank 4.0: shedding new light on drug metabolism. Nucleic Acids Res 42, D1091–D1097, doi: 10.1093/nar/gkt1068 (2014). 24203711 PMC3965102

[b36] Rose P. W. *et al.* The RCSB Protein Data Bank: views of structural biology for basic and applied research and education. Nucleic Acids Res 43, D345–D356, doi: 10.1093/nar/gku1214 (2015). 25428375 PMC4383988

[b37] Singh S. *et al.* PEPstrMOD: structure prediction of peptides containing natural, non-natural and modified residues. Biol Direct 10, 73, doi: 10.1186/s13062–015–0103–4 (2015). 26690490 PMC4687368

[b38] Kaur H., Garg A. & Raghava G. P. PEPstr: a de novo method for tertiary structure prediction of small bioactive peptides. Protein Pept Lett 14, 626–631 (2007). 17897087 10.2174/092986607781483859

[b39] Khoury G. A. *et al.* Forcefield_NCAA: ab initio charge parameters to aid in the discovery and design of therapeutic proteins and peptides with unnatural amino acids and their application to complement inhibitors of the compstatin family. ACS Synth Biol 3, 855–869, doi: 10.1021/sb400168u (2014). 24932669 PMC4277759

[b40] Khoury G. A., Thompson J. P., Smadbeck J., Kieslich C. A. & Floudas C. A. Forcefield_PTM: Charge and AMBER Forcefield Parameters for Frequently Occurring Post-Translational Modifications. J Chem Theory Comput 9, 5653–5674, doi: 10.1021/ct400556v (2013). 24489522 PMC3904396

[b41] Gfeller D., Michielin O. & Zoete V. SwissSidechain: a molecular and structural database of non-natural sidechains. Nucleic Acids Res 41, D327–D332, doi: 10.1093/nar/gks991 (2013). 23104376 PMC3531096

[b42] Yang J. *et al.* The I-TASSER Suite: protein structure and function prediction. Nat Methods 12, 7–8, doi: 10.1038/nmeth.3213 (2015). 25549265 PMC4428668

[b43] Kabsch W. & Sander C. Dictionary of protein secondary structure: pattern recognition of hydrogen-bonded and geometrical features. Biopolymers 22, 2577–2637, doi: 10.1002/bip.360221211 (1983). 6667333

[b44] O’Boyle N. M. *et al.* Open Babel: An open chemical toolbox. J Cheminform 3, 33, doi: 10.1186/1758–2946–3–33 (2011). 21982300 PMC3198950

[b45] Lazaro E. *et al.* Variable HIV peptide stability in human cytosol is critical to epitope presentation and immune escape. J Clin Invest 121, 2480–2492, doi: 10.1172/JCI44932 (2011). 21555856 PMC3104749

[b46] Lee V. Peptide and protein drug delivery. Vol. 4 (CRC Press, 1990).

[b47] Lin J. H. & Lu A. Y. Role of pharmacokinetics and metabolism in drug discovery and development. Pharmacol Rev 49, 403–449 (1997). 9443165

[b48] Adessi C. & Soto C. Converting a peptide into a drug: strategies to improve stability and bioavailability. Curr Med Chem 9, 963–978 (2002). 11966456 10.2174/0929867024606731

[b49] Gentilucci L., De Marco R. & Cerisoli L. Chemical modifications designed to improve peptide stability: incorporation of non-natural amino acids, pseudo-peptide bonds, and cyclization. Curr Pharm Des 16, 3185–3203 (2010). 20687878 10.2174/138161210793292555

[b50] Yamada A., Sasada T., Noguchi M. & Itoh K. Next-generation peptide vaccines for advanced cancer. Cancer Sci 104, 15–21, doi: 10.1111/cas.12050 (2013). 23107418 PMC7657262

