# Supplementary material for: PEPlife: A Repository of the Half-life of Peptides
Source: Sci Rep. 2016 Nov 7;6:36617. doi: 10.1038/srep36617 (PMC5098197; doi:10.1038/srep36617)
Supplement: Supplementary Information [file srep36617-s1.pdf]

PEPlife: A Repository of the Half-life of Peptides

Deepika Mathur[1], Satya Prakash[1,+,$], Priya Anand[1,+], Harpreet Kaur[1], Piyush Agrawal[1], Ayesha Mehta[1], Rajesh Kumar[1], Sandeep Singh[1], Gajendra P.S. Raghava[1,*]

[1]Bioinformatics Centre, CSIR-Institute of Microbial Technology, Chandigarh, India

[$]Present address- TIFR Centre for Interdisciplinary Sciences, Tata Institute of Fundamental Research, Narsingi, Hyderabad 500075, India

*Correspondence and requests for materials should be addressed to G.P.S.R. (email: raghava@imtech.res.in)

[+] These authors contributed equally to this work.

Supplementary Information:

Supplementary Table S1: Examples to show the effects of modifications on half-life of peptides. Details of these peptides with clickable links are available at PEPlife database (http://crdd.osdd.net/raghava/peplife).

PEPlife ID	Name	Sequence	N-terminal modification	C-terminal modification	Chemical Modification	Linear /Cyclic	Chirality	Half-life (seconds)	Tissue sample	Patent/ PMID
2866	GRF(1-29)-NH2 (Growth regulating factor)	YADAIFTNS YRKVLGQL SARKLLQDI MSR	Free	Amidation	None	Linear	L	372	Rat plasma	2896343
2867	[D-Ala2]-GRF(1-29)-NH2 (Growth regulating factor)	YaDAIFTNS YRKVLGQL SARKLLQDI MSR	Free	Amidation	None	Linear	Mix	396	Rat plasma	2896343
2868	[D-Asp3]-GRF(1-29)-NH2 (Growth regulating factor)	YAdAIFTNS YRKVLGQL SARKLLQDI MSR	Free	Amidation	None	Linear	Mix	354	Rat plasma	2896343
2869	lD-Asn8]-GRF(1-29)-NH2 (Growth regulating factor)	YADAIFTnS YRKVLGQL SARKLLQDI MSR	Free	Amidation	None	Linear	Mix	414	Rat plasma	2896343
2870	[D-Tyr10]-GRF(1-29)-NH2 (Growth regulating factor)	YADAIFTNS yRKVLGQL SARKLLQDI MSR	Free	Amidation	None	Linear	Mix	282	Rat plasma	2896343
2871	Ac-[D-Tyr1,D-AIa2]GRF(1-29)-NH2 (Growth regulating factor)	yaDAIFTNS YRKVLGQL SARKLLQDI MSR	Acetylation	Amidation	None	Linear	Mix	444	Rat plasma	2896343
1790	T-1249	WQEWEQKI TALLEQAQI QQEKNEYE LQKLDKW ASLWEWF	Free	Free	None	Linear	L	21240	Monkey blood	17640899
2215	T-1249	WQEWEQKI TALLEQAQI QQEKNEYE LQKLDKW ASLWEWF	Acetylation	Amidation	None	Linear	L	19980	Monkey blood	US6656906
2903	DSIP (Delta sleep inducing peptide)	WAGGDAS GE	Free	Free	None	Linear	L	434.4	Dog plasma	3768731
2906	D-Ala4-DSIP	WAGaDASG E	Free	Free	None	Linear	Mix	774	Dog plasma	3768731
2907	D-Ala4-DSIP-NH2	WAGaDASG E	Free	Amidation	None	Linear	Mix	612	Dog plasma	3768731
2905	D-Ala3-DSIP	WAaGDASG E	Free	Free	None	Linear	Mix	491.4	Dog plasma	3768731
2639	O-1(Oncocin)	VDKPPYLP RPRPPRRIY NR	Free	Amidation	None	Linear	L	1500	Mouse serum	21185160
2648	O-11 (Oncocin)	VDKPPYLP RPRPPRRIY Nr	Free	Amidation	None	Linear	Mix	9600	Mouse serum	21185160
2652	O-16 (Oncocin)	VDKPPYLP RPRPPRrIY NR	Free	Amidation	None	Linear	Mix	3480	Mouse serum	21185160
2660	O-24 (Oncocin)	VDKPPYLP	Free	Amidation	None	Linear	Mix	28800	Mouse	2118516

		RPRPPRrIY Nr							serum	0
2641	O-4 (Oncocin)	VDKPPYLP RPRPPRRIY NN	Free	Amidation	None	Linear	L	7200	Mouse serum	2118516 0
2650	O-14 (Oncocin)	VDKPPYLP RPRPPR-Orn-IYNR	Free	Amidation	Orn=Ornithin e	Linear	L	5400	Mouse serum	2118516 0
2659	O-23(Oncocin)	VDKPPYLP RPRPPR-Orn-IYN-Orn	Free	Amidation	Orn=Ornithin e	Linear	L	10560	Mouse serum	2118516 0
1006	Com1	RRWWRF	Free	Free	None	Linear	L	1800	Human serum	2084476 5
1012	Com2	RRWWRF	Free	Amidation	None	Linear	L	1800	Human serum	2084476 5
1018	Com3	RRWWRF	Acetylation	Free	None	Linear	L	3600	Human serum	2084476 5
1024	Com4	RRWWRF	Acetylation	Amidation	None	Linear	L	5400	Human serum	2084476 5
1030	Com5	RRWWRF	Free	Free	None	Cyclic (head-to-tail cycliza tion)	L	86400	Human serum	2084476 5
1039	Lfc7	RRWQWRM KKLG	Free	Free	None	Linear	L	1800	Human serum	2084476 5
1045	Lfc8	RRWQWRM KKLG	Free	Free	None	Cyclic (head-to-tail cycliza tion)	L	6600	Human serum	2084476 5
1094	iAβ5p-C1	LPFFD	Free	Amidation	None	Linear	L	300	Human plasma	1257883 0
1097	iAβ5p	LPFFD	Free	Free	None	Linear	L	900	Human plasma	1257883 0
1216	[Aib35]hGLP-1(7-36)NH2	HAEGTFTS DVSSYLEG QAAKEFIA WLVK-Aib-R	Free	Amidation	Aib=2-aminoisobuty ric acid	Linear	L	66240	Human plasma	2111459 9
1215	[Aib8]hGLP-1(7-36)NH2	H-Aib-EGTFTSDVS SYLEGQAA KEFIAWLV KGR	Free	Amidation	Aib=2-aminoisobuty ric acid	Linear	L	43560	Human plasma	2111459 9
1107	(Abu8)GLP-1	H-Abu-EGTFTSDVS SYLEGQAA KEFIAWLV KGR	Free	Free	Abu=2-aminobutyric acid	Linear	L	43200	Human plasma	1524686 9
2413	Octreotide	FCFWKTCT	Free	Free	None	Cyclic (C2-C7)	L	6780	Human serum	7911441
2475	Octreotide	fCFwKTCT	Free	Free	None	Cyclic (C2-C7)	Mix	5400	Human serum	8289671
1036	Com6	CRRWWRF C	Free	Amidation	None	Cyclic (C1-C8)	L	23400	Human serum	2084476 5
1042	Com7	CRRWWRF C	Acetylation	Amidation	None	Cyclic (C1-C8)	L	23400	Human serum	2084476 5
2161	Chimeric CNP-C	CFGLKLDRI GSMSGLGC VQQRKESK KPPAKLQP R	Free	Free	None	Cyclic (C1-C17)	L	901.8	Rat blood	EP22778 90A1
2167	Chimeric CNP-I	CFGLKLDRI GSMSGLGC VQQRKESK KPPAKLQP R	Free	Amidation	None	Cyclic (C1-C17)	L	870	Rat blood	EP22778 90A1
1265	GRF1-29	YADAIFTNS YRKVLGQL	Free	Amidation	None	Linear	L	900	Human plasma	1449970 7

		SARKLLQDI MSR								
1266	GRF-1PEGLys12	YADAIFTNS YRKVLGQL SARKLLQDI MSR	Free	Amidation	PEGylation on Lys12	Linear	L	36000	Human plasma	14499707
1268	GRF-1PEGLys21	YADAIFTNS YRKVLGQL SARKLLQDI MSR	Free	Amidation	PEGylation on Lys21	Linear	L	14400	Human plasma	14499707
1745	GLP-1	HAEGTFTS DVSSYLEG QAAKEFIA WLVKGR	Free	Amidation	None	Linear	L	156.6	Rat blood	15769092
1746	PEG2k-Lys-GLP-1	HAEGTFTS DVSSYLEG QAAKEFIA WLVKGR	Free	Amidation	Pegylation at Lysine	Linear	L	2001.6	Rat blood	15769092
1761	N-PEG/GLP-1	HAEGTFTS DVSSYLEG QAAKEFIA WLVKGR	Pegylation at His	Amidation	None	Linear	L	453600	Rat plasma	16586064
2725	TRI-1144	TTWEAWD RAIAEYAA RIEALLRAL QEQQEKNE AALREL	Acetylation	Amidation	None	Linear	L	15120	Rat plasma	25900863
2726	PEG-TRI-1144	TTWEAWD RAIAEYAA RIEALLRAL QEQQEKNE AALREL	Acetylation	Amidation	Conjugated with PEG(40KDa) through Lys-30	Linear	L	122400	Rat plasma	25900863
